# Evaluation of the rapid immunochromatographic ODK0501 assay for *Streptococcus pneumoniae* antigen detection with nasopharyngeal swabs: preliminary report

**DOI:** 10.1186/s40248-016-0060-5

**Published:** 2016-06-20

**Authors:** Shoji Suzuki, Naoki Nishimura, Torahiko Jinta, Yasuhiko Yamano, Genta Ishikawa, Yutaka Tomishima, Noboru Uchiyama, Naohiko Chohnabayashi

**Affiliations:** Division of Pulmonary Medicine, St. Luke’s International Hospital, 9-1, Akashi-cho, Chuo City, Tokyo 104-8560 Japan

**Keywords:** Immunochromatographic assay, Pneumonia, RAPIRUN® *S. pneumoniae*, Nasopharyngeal swab

## Abstract

**Background:**

Early identification and control of pathogenic bacteria are important in the treatment of pneumonia. Currently, two rapid antigen detection kits for pneumococcal pneumonia are available: one uses urine samples and the other, named RAPIRUN® *S. pneumoniae*, uses sputum samples. RAPIRUN® has shown high sensitivity with nasopharyngeal swab samples from pediatric patients. In this study, we investigated the performance of RAPIRUN® with nasopharyngeal swabs from adult patients.

**Methods:**

All adult patients diagnosed with pneumonia from November 2011 to April 2012 in St. Luke’s International hospital were included in this cross-sectional study. Single sputum, nasopharyngeal swab, and urine samples obtained from patients were investigated using a rapid antigen detection kit. Sputum and blood cultures were also evaluated. We compared the characteristics of pneumococcal pneumonia patients diagnosed using RAPIRUN with a nasopharyngeal swab to those patients diagnosed using other methods. Sensitivity and specificity were also calculated.

**Results:**

Seventeen out of 60 patients with pneumonia were diagnosed with pneumococcal pneumonia. In 4 out  of the 17 cases, a positive test result was obtained using RAPIRUN with a nasopharyngeal swab. The sensitivity and specificity were 23.5 and 100 %, respectively.

**Conclusion:**

RAPIRUN performed with nasopharyngeal swabs from adult patients exhibited lower sensitivity for the diagnosis of pneumococcal pneumonia than the other compared methods. The causative pathogen of pneumonia should be identified using not only sputum cultures or rapid antigen detection kits but also clinical features or gram staining of sputum.

## Background

Pneumonia was the fourth most common cause of death worldwide in 2012 [1], and it is a critical illness especially for the elderly because of increase in its incidence and severity [2]. The most common causative pathogen of pneumonia is *Streptococcus pneumonia*e, which causes a serious form of pneumonia [3, 4]. Although pneumococcal vaccination is an efficient prevention strategy against pneumococcal pneumonia, the vaccine does not cover all serotypes of *S. pneumoniae* [5]. Thus, early identification of the pathogenic bacteria and appropriate initial treatment remain important to an optimal outcome.

Currently, two rapid detection kits for *S. pneumoniae* antigen are available: BinaxNOW® *S. pneumoniae*, which uses urine samples, and an immunochromatographic ODK0501 assay named RAPIRUN® *S. pneumoniae*, which uses sputum and nasopharyngeal swab samples (hereafter referred to as BinaxNOW and RAPIRUN, respectively). It has been reported that the use of BinaxNOW with urine samples and RAPIRUN with sputum samples from adult pneumonia patients shows high efficiency in the diagnosis of pneumococcal pneumonia [6–10]. In addition, RAPIRUN performed with sputum samples shows higher sensitivity than BinaxNOW [9, 10]. However, RAPIRUN requires good quality sputum to achieve higher sensitivity [11], and the sensitivity of RAPIRUN might be lower in the elderly, who have difficulty coughing up sputum.

RAPIRUN has also been found to exhibit high sensitivity for pneumococcal pneumonia when used with nasopharyngeal swabs from pediatric patients [12]. However, the efficiency of RAPIRUN with nasopharyngeal swabs from adult patients has not been reported. In this study, we investigated the performance of RAPIRUN with nasopharyngeal swabs for the diagnosis of pneumococcal pneumonia in adult patients.

## Methods

### Patient population and diagnostic criteria

The St. Luke’s International Hospital Research Ethics Committee approved all aspects of this study (approval number 11-R127, approval date October 24, 2011). This was a cross-sectional study conducted at St. Luke’s International Hospital, Tokyo, Japan, from November 2011 to April 2012. Written informed consent was obtained from 60 consecutive patients who were over 20 years old and were hospitalized with a diagnosis of pneumonia.

Pneumonia was defined as new infiltrates on a chest radiograph with symptoms indicating lower respiratory tract infection, such as cough, sputum, fever, or dyspnea.

### Sample collection and microbiological investigation

Single sputum, urine, and nasopharyngeal swab samples were collected from the patients. Nasopharyngeal swab samples were collected by inserting swab into a nostril straight back along the floor of the nasal passage until reaching the posterior wall of the nasopharynx. Sputum and nasopharyngeal swab samples were analyzed using RAPIRUN® (Otsuka Pharmaceutical Co., Ltd., Tokyo, Japan). We also analyzed the urine samples using the BinaxNOW® rapid antigen detection kit (Binax Inc., Scarborough, ME, USA). Sputum, nasopharyngeal, and blood cultures were also evaluated.

### Using RAPIRUN®

We used swabs to collect sputum and nasopharyngeal samples, and the extraction solutions were added to the test kit. Two lines indicated a positive result, i.e., the test and control, and a negative result showed only the control line. This test required approximately 25 min in total.

### Data collection

In addition to bacterial information, we collected clinical data, such as those pertaining to age, sex, smoking habits, existing underlying diseases, residence, history of pneumococcal vaccination, prior antibiotic therapy, vital signs, medication, laboratory testing, and chest radiographs, at admission. We also calculated the Patient Outcomes Research Team (PORT) score.

### Diagnosis of pneumococcal pneumonia

Sputum culture is the most commonly used reference standard for the detection of the causative pneumonia pathogens, but both the sensitivity and specificity are not sufficient [13, 14]. We determined the causative pneumonia pathogen using a combination of multiple diagnostic test results, that is the results for sputum culture, blood culture, and a rapid antigen detection kit (RAPIRUN with sputum and BinaxNOW with urine). A diagnosis of pneumococcal pneumonia was made if any test results showed evidence of *S. pneumoniae*.

### Analysis

We compared the characteristics of patients with pneumococcal pneumonia with those of patients with pneumonia caused by other pathogens using Fisher’s exact probability test or Mann-Whitney’s U test (two tailed, with a *p* of < 0.05 indicating a significant difference).

We also compared the patients with pneumococcal pneumonia diagnosed using RAPIRUN with a nasopharyngeal swab with those diagnosed using other methods. The sensitivity and specificity of RAPIRUN performed with a nasopharyngeal swab for diagnosing pneumococcal pneumonia were also calculated.

## Results

We collected samples from 60 consecutive inpatients with pneumonia. Due to anuria in cases of end-stage renal failure, urine samples were obtained from only 58 patients. The average age of all patients was 81 (28–96) years, and more than half of the patients were male (58.3 %) and smokers (55.0 %). Commonly, observed underlying diseases included diabetes mellitus, chronic obstructive pulmonary disease, and malignant neoplasm. Thirteen out of 60 (21.7 %) patients had received the 23-valent pneumococcal polysaccharide vaccine.

Seventeen out of 60 (28.3 %) patients had been diagnosed with pneumococcal pneumonia. For the 17 cases of pneumococcal pneumonia, 14 cases were positive using BinaxNOW, 12 cases were positive using RAPIRUN with sputum samples, 10 cases were positive for *S. pneumoniae* in the sputum culture, and one case was positive for *S. pneumoniae* in the blood culture. Table 1 lists the patients’ characteristics. C-reactive protein was significantly higher in patients with pneumococcal pneumonia than in patients with pneumonia caused by other bacteria. The proportion of patients who received a pneumococcal vaccine was lower in patients with pneumococcal pneumonia, but this difference was not significant.

**Table 1:** Characteristics of pneumonia patients

	Total (*n* = 60)		Pneumococcal pneumonia (*n* = 17)		Pneumonia caused by other bacteria (*n* = 43)		*P*
Age, years, median (range)	81	(28–96)	78	(28–96)	82	(40–96)	0.45
Male sex, n (%)	35	(58.3)	7	(41.2)	28	(68.3)	0.15
Nursing home residents, n (%)	8	(13.3)	0	0	8	(19.5)	0.091
Smoking history, n (%)	33	(55.0)	7	(41.2)	26	(63.4)	0.25
Patients with underlying disease							
Diabetes mellitus, n (%)	14	(23.3)	2	(11.8)	12	(29.3)	0.31
Bronchial asthma, n (%)	12	(20.0)	3	(17.6)	9	(22.0)	1.00
COPD^a^, n (%)	14	(23.3)	3	(17.6)	11	(26.8)	0.74
Liver cirrhosis, n (%)	0	(0.0)	0	(0.0)	0	(0.0)	-
Renal failure, n (%)	10	(16.7)	2	(11.8)	8	(19.5)	0.71
Heart failure, n (%)	7	(11.7)	1	(5.9)	6	(14.6)	0.66
Malignant neoplasm, n (%)	19	(31.7)	4	(23.5)	15	(36.6)	0.54
Patients vaccinated against *S. pneumoniae*, n (%)	13	(21.7)	1	(5.9)	12	(29.3)	0.087
Patients with prior antibiotic therapy, n (%)	23	(38.3)	4	(23.5)	19	(46.3)	0.16
Body temperature, °C, median (range)	37.6	(35.6–40.2)	37.8	(36.3–39.9)	37.6	(35.6–40.2)	0.71
White blood cells, /μL, median (range)	10,850	(2000–30,700)	11,900	(6100–30,700)	10,700	(2000–25,100)	0.52
C-reactive protein, mg/dL, median (range)	12.23	(0.12–40.73)	15.11	(0.92–36.7)	12.14	(0.12–40.73)	0.046
PORT^b^ score, points, median (range)	111.5	(32–236)	87	(38–236)	124	(32–186)	0.10

^a^Chronic obstructive pulmonary disease

^b^Pneumonia Patient Outcomes Research Team

### Results of RAPIRUN with nasopharyngeal swabs

Out of the 60 patients who participated in this study, only four patients showed positive test results when using RAPIRUN with nasopharyngeal swabs. The characteristics of these four patients and the results of the microbiological investigations are shown in Tables 2 and 3, respectively. All four cases were diagnosed as pneumococcal pneumonia using BinaxNOW performed with a urine sample or RAPIRUN performed with a sputum sample. Interestingly, only two patients had positive nasopharyngeal culture for *S. pneumoniae* among four cases with positive result of RAPIRUN with a nasopharyngeal swab. There was an insufficient number of patients; however, the sensitivity and specificity of RAPIRUN with a nasopharyngeal swab were calculated as 23.5 and 100 %, respectively.

**Table 2:** Clinical characteristics of patients with positive results using RAPIRUN with nasopharyngeal swabs

	All pneumococcal pneumonia patients (*n* = 17)		Patients with positive results using RAPIRUN with nasopharyngeal swabs (*n* = 4)	
Age, years, median (range)	78	(28–96)	79.5	(76–87)
Male sex, n (%)	7	(41.2)	0	(0.0)
Nursing home residents, n (%)	0	(0.0)	0	(0.0)
Smoking history, n (%)	7	(41.2)	0	(0.0)
Patients with underlying disease				
Diabetes mellitus, n (%)	2	(11.8)	0	(0.0)
Bronchial asthma, n (%)	3	(17.6)	0	(0.0)
COPD^a^, n (%)	3	(17.6)	0	(0.0)
Liver cirrhosis, n (%)	0	(0.0)	0	(0.0)
Renal failure, n (%)	2	(11.8)	0	(0.0)
Heart failure, n (%)	1	(5.9)	0	(0.0)
Malignant neoplasm, n (%)	4	(23.5)	1	(25.0)
Patients vaccinated against *S. pneumoniae*, n (%)	1	(5.9)	1	(25.0)
Patients with prior antibiotic therapy, n (%)	4	(23.5)	0	(0.0)
Body temperature, °C, median (range)	37.8	(36.3–39.9)	39.1	(38.3–39.4)
White blood cells, /μL, median (range)	11,900	(6100–30,700)	9000	(6600–13,300)
C-reactive protein, mg/dL, median (range)	15.11	(0.92–36.7)	17.5	(14.1–34.7)
PORT^b^ score, points, median (range)	87	(38–236)	92.5	(86–101)

^a^Chronic obstructive pulmonary disease

^b^Pneumonia Patient Outcomes Research Team

**Table 3:** Results of sputum culture and antigen detection using urine, sputum, and nasopharyngeal swab samples for 17 patients diagnosed with pneumococcal pneumonia

No.	Sputum culture	Urinary antigen	Sputum antigen	Nasopharyngeal swabs	Nasopharyngeal culture
1	*Streptococcus pneumoniae*	-	+	-	*S. pneumoniae*
2	*Klebsiella pneumoniae*	-	+	-	*K. pneumoniae*
3	*Staphylococcus aureus*	+	+	+	*S. aureus*
4	*Moraxella*	+	-	-	*α Streptococcus*
5	*S. pneumoniae*	+	+	-	*S. pneumoniae*
6	*Neisseria spp*	+	-	-	*α Streptococcus*
7	*S. pneumoniae*	+	+	+	*S. pneumoniae*
8	*S. pneumoniae*	-	+	-	*α Streptococcus*
9	*S. pneumoniae*	+	+	+	*S. pneumoniae*
10	*S. pneumoniae, Escherichia coli*	+	+	-	*S. aureus*
11	Not detected	+	-	-	*S. aureus*
12	*S. pneumoniae*	+	+	-	*α Streptococcus*
13	*S. pneumoniae*	+	+	-	*α Streptococcus*
14	Not detected	+	-	-	*S. aureus*
15	*S. pneumoniae*	+	+	-	*α Streptococcus*
16	Not detected	+	-	-	*α Streptococcus*
17	*S. pneumoniae*	+	+	+	*α Streptococcus*

## Discussion

We diagnosed 17 patients as having pneumococcal pneumonia, and only four of these patients showed positive results when using RAPIRUN with nasopharyngeal swabs, suggesting low sensitivity, although RAPIRUN with sputum samples previously showed high sensitivity [10]. Iwata et al. [12] reported that RAPIRUN exhibited high sensitivity (33/50, 66 %) and specificity (55/55, 100 %) when used with nasopharyngeal swabs in children (under 15 years of age). The results of the present study suggest that RAPIRUN with nasopharyngeal swabs should not be used for the diagnosis of pneumococcal pneumonia in adult patients. The discrepancy of result between RAPIRUN with nasopharyngeal swab and nasopharyngeal culture might suggest that nasopharyngeal swab sample from adult patients with pneumonia are not useful.

In this study, 17 patients were diagnosed with pneumococcal pneumonia using BinaxNOW, RAPIRUN with a sputum sample, or sputum culture. However, these cases may include false positive cases, for example, due to colonization or cross-reaction of antigens, and false negative cases. Because it is very difficult to detect the causative pathogen of pneumonia, clinical data, gram staining of sputum, or radiographic patterns should be considered in addition to sputum culture and rapid antigen detection kits. It might not be suitable to base a diagnosis only on the results of RAPIRUN with a nasopharyngeal swab in adult patients because of low sensitivity.

This study only included inpatients with fairly severe cases of pneumonia. Further studies targeting not only inpatients but also outpatients with pneumonia are required. The small number of pneumococcal pneumonia patients is also a limitation of this study. Further investigation with larger number of participants would be required.

## Conclusions

In conclusion, RAPIRUN exhibited low sensitivity for the diagnosis of pneumococcal pneumonia when used with nasopharyngeal swab samples from adult patients. The causative pathogen of pneumonia should be identified using not only sputum culture or rapid antigen detection kits but also clinical features or gram staining of sputum.

## Ethics approval and consent to participate

The St. Luke’s International Hospital Research Ethics Committee approved all aspects of this study (approval number 11-R127, approval date October 24, 2011). Written informed consent was obtained from all participants.

## Abbreviations

COPD: chronic obstructive pulmonary disease
PORT: Pneumonia Patient Outcomes Research Team
